# Haploidentical, matched-related, and matched-unrelated hematopoietic cell transplant for acute leukemias in the early years of haploidentical transplant implementation in a developing country with a large unrelated donor registry

**DOI:** 10.3389/fonc.2025.1584631

**Published:** 2025-04-15

**Authors:** Adriana Seber, Leonardo Javier Arcuri, Vergilio Rensi Colturato, Mair Pedro Souza, Yana Augusta Novis Zogbi, Vaneuza Funke, Decio Lerner, Maria Cristina Macedo, Liane Daudt, Mariana Nassif Kerbauy, Victor Gottardello Zecchin, Fernando Barroso Duarte, Ricardo Rabello Chiattone, Rodolfo Daniel de Almeida Soares, Gustavo Bettarello, Antonio Vaz de Macedo, Eduardo Paton, Tatiana Dias Marconi Monteiro, Jayr Schmidt Filho, Claudia Caceres Astigarraga, Phillip Scheinberg, Afonso Celso Vigorito, Carmen Silvia Vieitas Vergueiro, Anderson Simione, Shahrukh Hashmi, Wael Saber, Jinalben Patel, Carmem Maria Sales Bonfim, Marcelo Pasquini, Mary Evelyn Flowers, Nelson Hamerschlak

**Affiliations:** ^1^ Bone Marrow Transplant Department, Hospital Samaritano, Sao Paulo, Brazil; ^2^ Bone Marrow Transplant Department, Hospital Israelita Albert Einstein, Sao Paulo, Brazil; ^3^ Bone Marrow Transplant Department, Hospital Amaral Carvalho, Jau, Brazil; ^4^ Oncohematology Department, Hospital Sirio-Libanes, Sao Paulo, Brazil; ^5^ Bone Marrow Transplant Department, Universidade Federal do Parana, Curitiba, Brazil; ^6^ Bone Marrow Transplant Department, Instituto Nacional de Cancer, Rio de Janeiro, Brazil; ^7^ Bone Marrow Transplant Team, Bio Sana’s Servicos Medicos, Sao Paulo, Brazil; ^8^ Bone Marrow Transplant Department, Universidade Federal do Rio Grande do Sul, Porto Alegre, Brazil; ^9^ Pediatric Bone Marrow Transplant, Beneficiencia Portuguesa de Sao Paulo, Sao Paulo, Brazil; ^10^ Hematology, Oncology, and Bone Marrow Transplant Department, Universidade Federal do Ceara, Fortaleza, Brazil; ^11^ Bone Marrow Transplant Department, Universidade Federal do Rio Grande do Norte, Natal, Brazil; ^12^ Bone Marrow Transplant Department, Hospital DF Star Rede D’or, Brasilia, Brazil; ^13^ Hematology Department, Hospital da Policia Militar de Minas Gerais, Belo Horizonte, Brazil; ^14^ Bone Marrow Transplant and Cellular Therapy Unit, Cancer Center Oncoclinicas, Nova Lima, Brazil; ^15^ Bone Marrow Transplant Unit, Centro de Hematologia de Hemoterapia de Santa Catarina, Florianopolis, Brazil; ^16^ Hematology, Bone Marrow Transplant, and Cellular Therapy Department, A. C. Camargo Cancer Center, Sao Paulo, Brazil; ^17^ Hematology and Oncology Department, Beneficiencia Portuguesa de Sao Paulo, Sao Paulo, Brazil; ^18^ Hematology, Hemotherapy, and Bone Marrow Transplant Center, Universidade Estadual de Campinas, Campinas, Brazil; ^19^ Associacao de Medula Ossea do Estado de Sao Paulo, Sao Paulo, Brazil; ^20^ Center for International Blood and Marrow Transplant Research, Medical College of Wisconsin, Milwaukee, WI, United States; ^21^ Duke University, Transplant and Cellular Therapy Department, Durham, NC, United States; ^22^ Fred Hutch Cancer Center, Clinical Research Division, Seattle, WA, United States

**Keywords:** hematopoietic cell transplantation, survival bias, acute lymphoblastic leukemia, haploidentical transplantation, unrelated donors, matched-related donors, posttransplant cyclophosphamide

## Abstract

**Introduction:**

Over the last decades, the donor network for hematopoietic cell transplantation (HCT) has grown exponentially, including unrelated and haploidentical (Haplo) donors. This study aimed to describe HCT outcomes with MSD, Haplo, and matched unrelated donors (MUD) in an early period of Haplo with posttransplant cyclophosphamide in a developing country with a large unrelated donor registry.

**Methods:**

This study was conducted in collaboration with the CIBMTR. We included patients with acute leukemias undergoing HCT between 2014-2018.

**Results:**

With 595 patients, 2-year overall survival (OS) was 69% for the MSD, 65% for the Haplo, and 71% for MUD (p=0.24) in CR1, confirmed in multivariable analysis. Relapse rate was lower for MUD (HR=0.35, p=0.0005) than MSD in patients with CR2+, leading to higher OS. Relapse was also higher with Haplo compared with MUD (HR=2.06, p=0.03).

**Discussion:**

Only survival bias can explain these findings in CR2+, suggesting some high-risk MUD patients, in which HCT timing is crucial, may not achieve HCT. Alternative donors were associated with higher non-relapse mortality, while PTCy-based Haplo offered the best protection against chronic graft-versus-host disease. Our study suggests Haplo and MUD are acceptable options for patients lacking MSD in developing countries like ours.

## Highlights

- Both Haplo and MUD are acceptable alternatives for those lacking a MSD.
- Additional analyses suggest that some high-risk patients with MUD may not achieve HCT.
- Survival bias and residual confounding may inflate MUD outcomes compared with Haplo.
- cGVHD was not different between MUD and Haplo.
- This study is the first in a developing country to use the CIBMTR’s resources and expertise to analyze local hematopoietic cell transplant results. We have found that, in a developing country with a large donor registry (the REDOME), the results of haploidentical transplantation are not inferior to matched donors in patients in CR1. We have found evidence that the observational comparison has inherent survival bias, limited to patients in CR2+, and neither survival bias nor residual confounding that can’t be controlled in the analysis.

## Introduction

Over the past few decades, the donor network for hematopoietic cell transplantation (HCT) has grown exponentially, making the procedure feasible for a significant portion of those without a suitable matched sibling donor (MSD).

In recent decades, the Brazilian government has invested in the REDOME (Registro Brasileiro de Doadores Voluntarios de Medula Ossea; REDOME is the Brazilian governmental agency where the Brazilian marrow donor registry is located) (1), the world’s third-largest bone marrow donor registry. Unrelated donor transplantation is recognized as an established procedure in Brazil. However, the utilization of unrelated donors through REDOME seems lower than that of international registries from developed countries (Brazilian data available at www.abto.org.br), and the reasons for this disparity are unclear. Consequently, since the introduction of haploidentical transplantation (Haplo) with posttransplant cyclophosphamide (PTCy) in 2008 by Luznik et al. (2), Brazilian centers have shifted toward this transplantation approach. Haploidentical transplantation using the PTCy platform began to be routinely performed in Brazil in 2012. The objective of the current study was to describe major HCT outcomes using MSD, matched unrelated donors (MUD), and Haplo donors in an early period to establish a benchmark assessment of this approach in developing countries.

## Methods

### Data sources

The CIBMTR (Center for International Blood and Marrow Transplant Research) is a research collaboration between the National Marrow Donor Program/Be The Match and the Medical College of Wisconsin. It comprises a voluntary working group of more than 360 participating centers worldwide that contribute detailed data on cellular therapies (https://cibmtr.org/CIBMTR/About/Our-Impact/Our-Centers). The Sociedade Brasileira de Transplante the Medula Ossea e Terapia Celular (Brazilian Society for Bone Marrow Transplantation and Cellular Therapy, SBTMO) and the CIBMTR established an agreement to use the CIBMTR structure as the framework for the Brazilian HCT Registry. The study was approved by the Ethics Committees (CAAE 78575317.6.1001.0071) of the participating centers (**Supplementary Table 1**) and by the International Studies Working Committee from the CIBMTR (HS-1904).

### Eligibility

We included all adult and pediatric patients undergoing their first allogeneic HCT in Brazil between January 1, 2014, and December 31, 2018, who were reported to the CIBMTR for acute lymphoblastic leukemia (ALL) or acute myeloid leukemia (AML) in complete remission with an MSD, HLA 8/8 MUD, or Haplo donor. Only Brazilian centers reporting to the CIBMTR were considered. The SBTMO designed this study in collaboration with the CIBMTR.

### Outcomes and definitions

Overall survival (OS) was the time from HCT to death from any cause. Non-relapse mortality (NRM) was the time from HCT to death without relapse or progression, treating relapse as a competing risk. Disease-free survival (DFS) refers to the time from HCT to relapse, progression, or death. Data on acute GVHD were largely unavailable, so this analysis did not include them. Chronic graft-versus-host disease (GVHD) was diagnosed using standard criteria.

### Statistical analysis

Patients’ characteristics were presented as median and interquartile range, or as number and percentages. The study’s primary objective was to describe OS after HCT performed in Brazil with MSD, MUD, and Haplo donors. The secondary objectives included a comparative analysis of OS, NRM, DFS, relapse, and GVHD among the HCT donor type groups.

Survival and cumulative incidence curves were constructed using the Kaplan-Meier and Gray methods, compared with the log-rank and Gray tests, respectively, and reported at the 2-year time point. Uni- and multivariable analyses were conducted with the Cox model. Results are expressed as hazard ratios (HR) and 95% confidence intervals (95% CI). Variables tested in multivariable analyses included patient age (by decade and ≥ 18 years of age *vs*. younger), patient/donor gender combination, HCT comorbidity index (0-2 or >3), Karnofsky performance score (<90 *vs*. 90-100), diagnosis (AML or ALL), disease status at HCT (first or second remission, or refractory/primary induction failure), donor type (MSD, MUD, Haplo), graft source (marrow or peripheral blood), conditioning regimen (myeloablative, non-myeloablative, or reduced intensity, **Supplementary Table 2**). Model selection was based on the lowest Akaike Information Criterion (AIC) using the complete cases database, which was then reported alongside the actual database. Interactions were tested, adding interaction terms to the Cox model. Anti-thymocyte globulin (ATG) and PTCy were predominantly used in MUD and Haplo HCT, respectively, and were therefore not tested in the multivariate analysis. CMV status was not tested in the multivariable models due to the very few patient-donor pairs that were negative for cytomegalovirus (CMV). Analyses were stratified by remission status. All analyses were conducted with R, R Foundation for Statistical Computing, Vienna, Austria, version 4.3.2.

## Results

Of the 595 patients included in this study, 338 (57%) had an MSD, 116 (24%) had a Haplo, and 141 (19%) had a MUD, with median follow-up times of 46, 29, and 36 months, respectively. Patient characteristics are shown in **Table 1**. In summary, recipients of MUD or Haplo in CR2+ transplants were younger than those receiving MSD, with a median of 12 and 16 years, respectively. The oldest groups were Haplo and MSD in CR1 (median of 37 and 36 years). Haplo recipients were less likely to have undergone a myeloablative conditioning (MAC) regimen, especially in CR1, and ATG-containing GVHD prophylaxis was strongly associated with MUD, reflecting local practices.

**Table 1:** Patients’ characteristics according to HCT donor type.

**Disease status**	Haplo		MSD		MUD		P value
	**CR1**	**CR2+**	**CR1**	**CR2+**	**CR1**	**CR2+**	
**Total**	60	56	248	90	79	62	
**Median age (IQR)**	36.3 (21.6,52.6)	12 (8.1,22)	36.8 (22.9,48.4)	23.7 (14.3,37.4)	22.1 (12.5,40.9)	15.8 (9.6,30.1)	< 0.001
**Children < 18 y/o**	8 (13.3%)	37 (66.1%)	29 (11.7%)	36 (40%)	28 (35.4%)	35 (56.5%)	< 0.001
**Female sex**	29 (48.3%)	28 (50%)	108 (43.5%)	40 (44.4%)	41 (51.9%)	22 (35.5%)	0.435
**Sex match**							0.022
** F->M**	9 (15%)	11 (19.6%)	69 (27.8%)	22 (24.4%)	8 (10.3%)	14 (22.6%)	
**Others**	51 (85%)	45 (80.4%)	179 (72.2%)	68 (75.6%)	70 (89.7%)	48 (77.4%)	
**HCT-CI**							0.263
** 0-2**	52 (86.7%)	52 (92.9%)	232 (94.3%)	80 (88.9%)	73 (92.4%)	59 (95.2%)	
** 3+**	8 (13.3%)	4 (7.1%)	14 (5.7%)	10 (11.1%)	6 (7.6%)	3 (4.8%)	
**KPS**							0.509
** 90-100**	50 (83.3%)	51 (92.7%)	215 (87.4%)	75 (83.3%)	67 (84.8%)	56 (90.3%)	
** <90**	10 (16.7%)	4 (7.3%)	31 (12.6%)	15 (16.7%)	12 (15.2%)	6 (9.7%)	
**Disease**							0.022
** AML**	33 (55%)	25 (44.6%)	147 (59.3%)	43 (47.8%)	31 (39.2%)	29 (46.8%)	
** ALL**	27 (45%)	31 (55.4%)	101 (40.7%)	47 (52.2%)	48 (60.8%)	33 (53.2%)	
**Female donor**	19 (31.7%)	22 (39.3%)	120 (48.4%)	48 (53.3%)	21 (26.9%)	20 (32.3%)	< 0.001
**Graft source**							0.381
** BM**	34 (56.7%)	38 (67.9%)	159 (64.1%)	64 (71.1%)	47 (59.5%)	36 (58.1%)	
** PBSC**	26 (43.3%)	18 (32.1%)	89 (35.9%)	26 (28.9%)	32 (40.5%)	26 (41.9%)	
**CMV**							0.116
** Neg/Neg**	4 (6.9%)	3 (5.5%)	7 (2.9%)	5 (5.8%)	1 (1.3%)	0 (0%)	
** Any Pos**	54 (93.1%)	52 (94.5%)	236 (97.1%)	81 (94.2%)	78 (98.7%)	62 (100%)	
**Conditioning**							< 0.001
** MAC**	32 (53.3%)	43 (76.8%)	211 (85.4%)	82 (91.1%)	69 (87.3%)	59 (95.2%)	
** NMA**	7 (11.7%)	6 (10.7%)	12 (4.9%)	0 (0%)	1 (1.3%)	0 (0%)	
** RIC**	21 (35%)	7 (12.5%)	24 (9.7%)	8 (8.9%)	9 (11.4%)	3 (4.8%)	
**ATG**	1 (1.7%)	1 (1.8%)	19 (7.7%)	5 (5.6%)	73 (92.4%)	61 (98.4%)	< 0.001
**Median follow-up (IQR)**	24.9 (12.5,37)	35.5 (12,46.7)	46.8 (26.8,60.8)	41.1 (24.4,57.8)	31.7 (22,50.4)	43.6 (24.2,58.5)	

**Figure 1:**
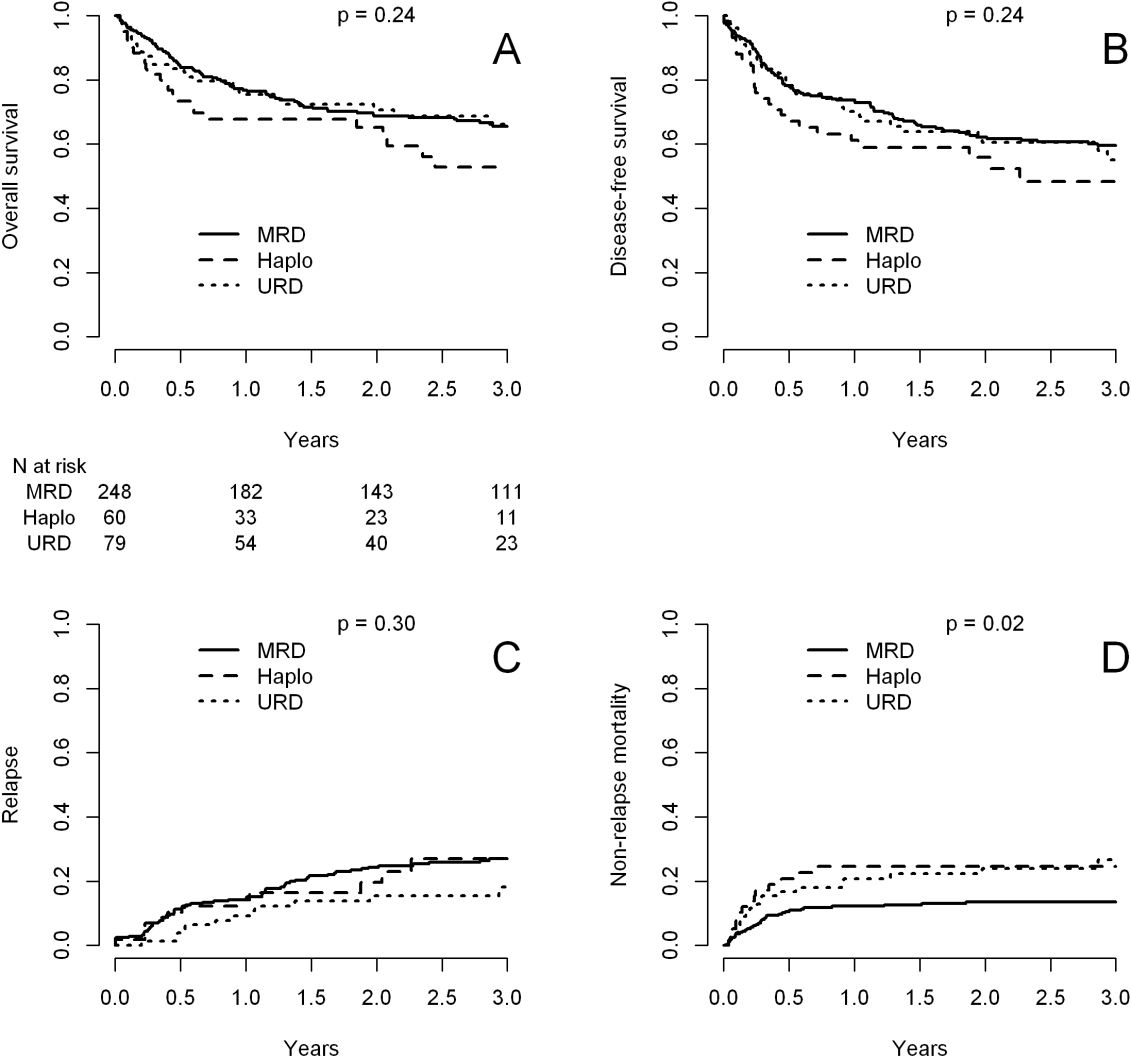
Transplant outcomes after transplant with matched sibling donors (MSD), haploidentical donors (Haplo) and matched unrelated donors (MUD) in patients in CR1. **(A)** Overall survival; **(B)** disease-free survival; **(C)** cumulative incidence of relapse; **(D)** cumulative incidence of non-relapse mortality.

**Figure 2:**
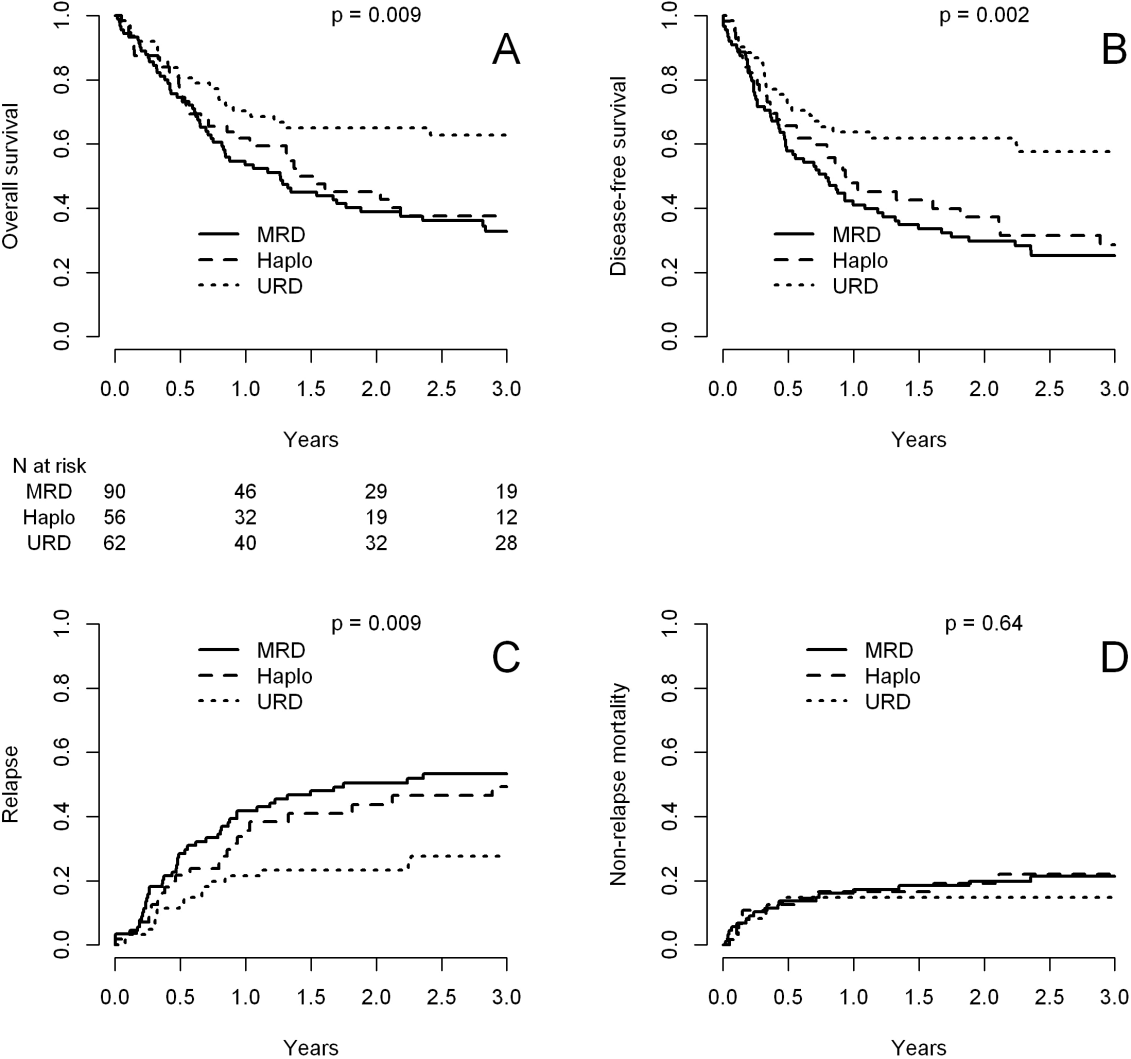
Transplant outcomes after transplant with matched sibling donors (MSD), haploidentical donors (Haplo) and matched unrelated donors (MUD) in patients in CR2+. **(A)** Overall survival; **(B)** disease-free survival; **(C)** cumulative incidence of relapse; **(D)** cumulative incidence of non-relapse mortality.

**Figure 3:**
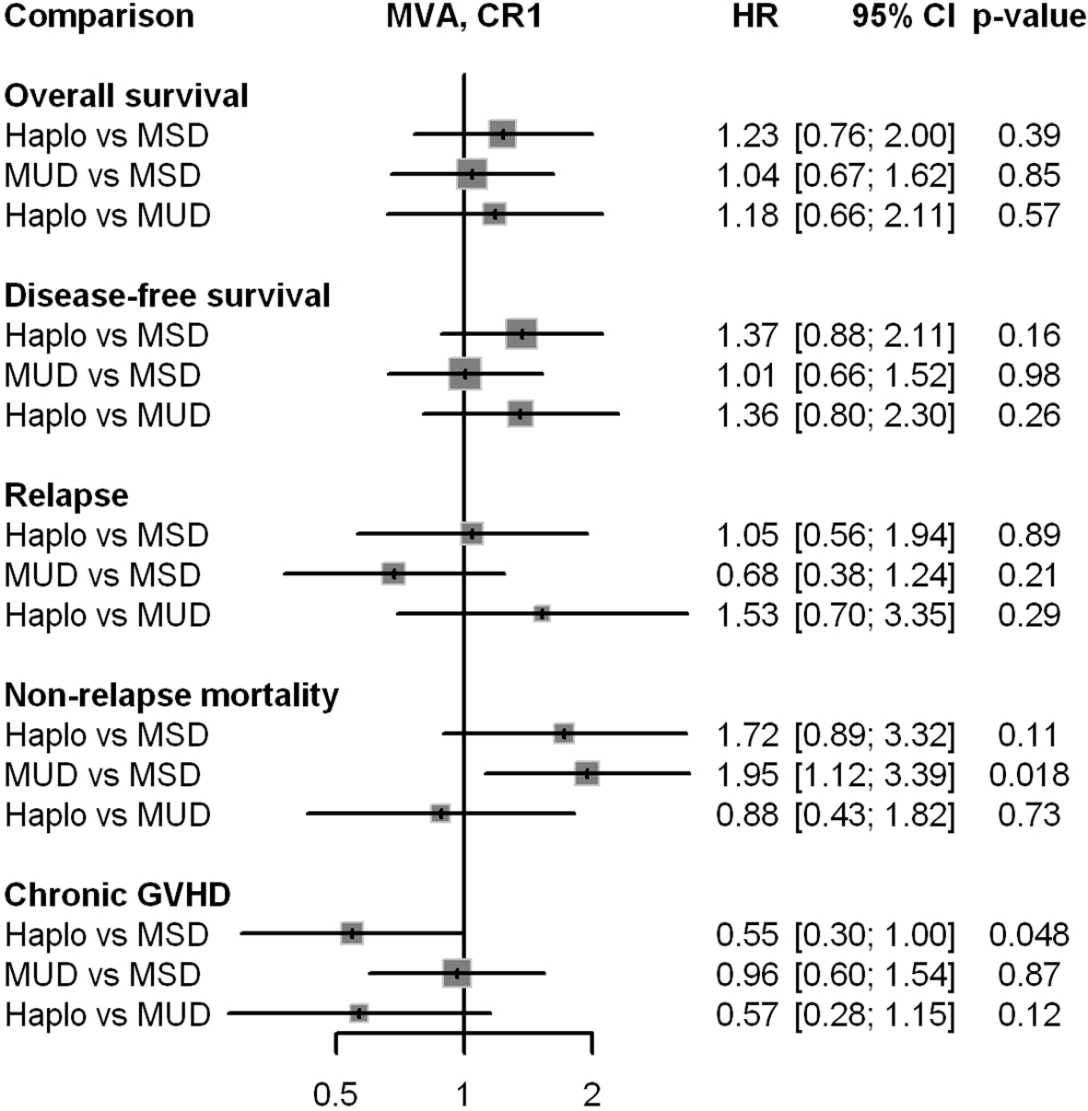
Graphical representation of the impact of donor type in multivariable analyses for all major outcomes in patients in CR1. HR, hazard ratio; Haplo, haploidentical HCT; MSD, matched-sibling donor; MUD, matched unrelated donor; GVHD, graft-versus-host disease; MVA, multivariable analysis.

**Figure 4:**
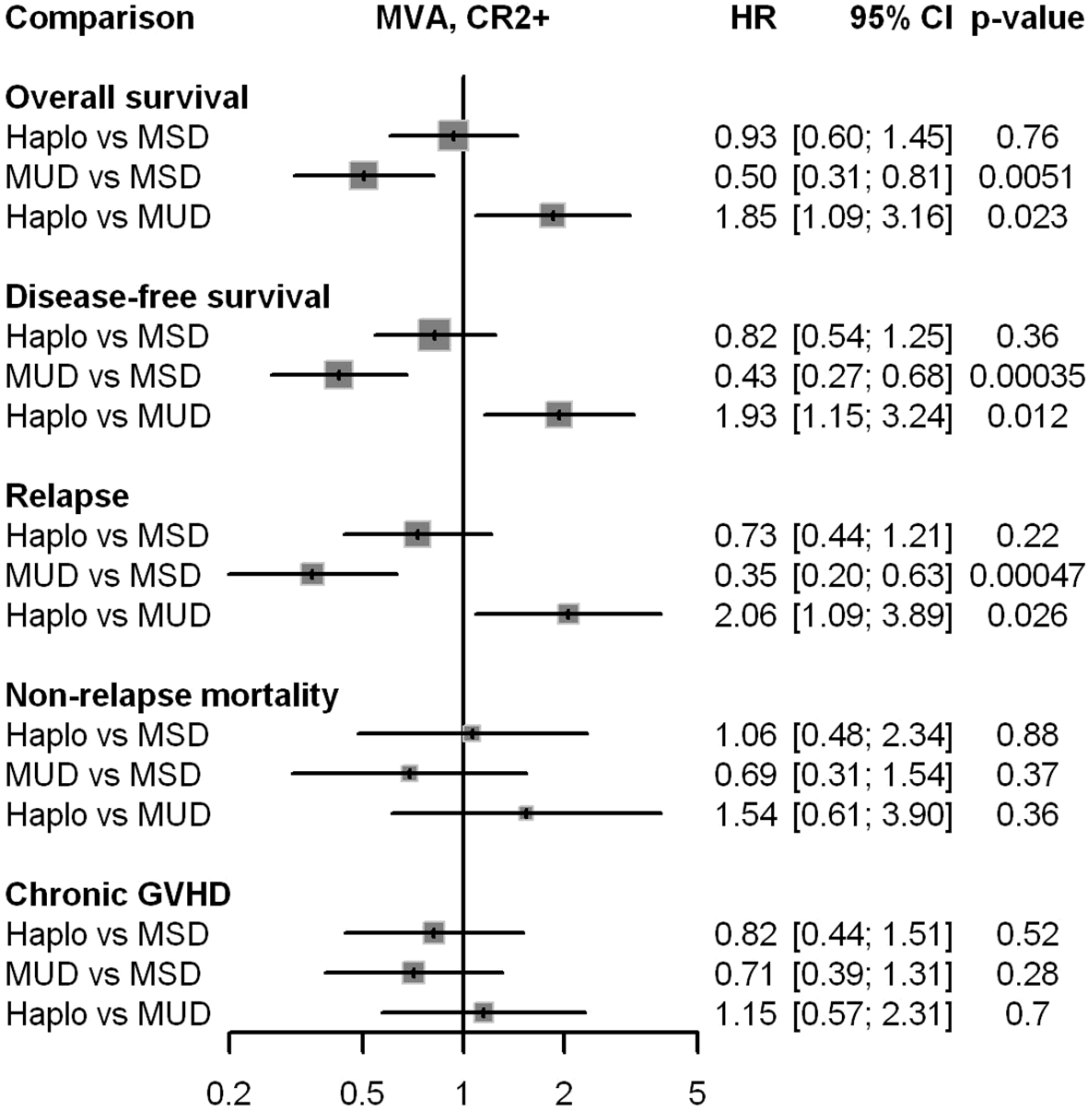
Graphical representation of the impact of donor type in multivariable analyses for all major outcomes in patients in CR2+. HR, hazard ratio; Haplo, haploidentical HCT; MSD, matched-sibling donor; MUD, matched unrelated donor; GVHD, graft-versus-host disease; MVA, multivariable analysis.

**Table 2** presents major HCT outcomes categorized by disease status and donor type groups. Multivariable models are detailed in **Table 3**. **Figures 1** and **2** provide the Kaplan-Meier and cumulative incidence curves for CR1 and CR2+, respectively. Graphical representations of the multivariable analyses are in **Figures 3** and **4**. **Supplementary Table 3** contains the other variables included in each multivariable Cox model.

**Table 2:** Univariable analyses of overall survival, non-relapse mortality, relapse, disease-free survival, and chronic graft-versus-host disease.

2y-outcome	CR1				CR2+			
	MSD	Haplo	MUD	p	MSD	Haplo	MUD	p
**Overall survival**	69%	65%	71%	0.24	39%	45%	65%	0.0085
**Disease-free survival**	62%	56%	61%	0.24	30%	37%	62%	0.0018
**Relapse**	24%	20%	15%	0.30	50%	44%	23%	0.0089
**Non-relapse mortality**	13%	25%	24%	0.017	20%	19%	15%	0.64
**Chronic GVHD**	44%	23%	31%	0.013	34%	32%	28%	0.76

MSD, matched-sibling donor; Haplo, haploidentical donor; MUD, matched-unrelated donor; GVHD, graft-versus-host disease; CR1, first complete remission; CR2+, subsequent remission.

**Table 3:** Multivariable analyses of overall survival, non-relapse mortality, relapse, disease-free survival, and chronic graft-versus-host disease.

Outcome, strata	HR	Haplo 95%CI	p	HR	MUD 95%CI	p
CR1						
** Overall survival**	1.23	0.76-2.00	0.39	1.04	0.67-1.62	0.85
** Disease-free survival**	1.37	0.88-2.11	0.16	1.01	0.66-1.52	0.98
** Relapse**	1.05	0.56-1.94	0.89	0.68	0.38-1.24	0.21
** Non-relapse mortality**	1.72	0.89-3.32	0.11	1.95	1.12-3.39	0.018
** Chronic GVHD**	0.55	0.300-1.00	0.048	0.96	0.60-1.54	0.87
CR2+						
** Overall survival**	0.93	0.60-1.45	0.76	0.50	0.31-0.81	0.0051
** Disease-free survival**	0.82	0.54-1.25	0.36	0.43	0.27-0.68	0.00035
** Relapse**	0.73	0.44-1.21	0.22	0.35	0.20-0.63	0.00047
** Non-relapse mortality**	1.06	0.48-2.34	0.88	0.69	0.31-1.54	0.37
** Chronic GVHD**	**0.82**	**0.44-1.51**	**0.52**	**0.71**	**0.39-1.31**	**0.28**

Reference category is matched-sibling donor; Haplo, haploidentical donor; MUD, matched-unrelated donor; GVHD, graft-versus-host disease; CR1, first complete remission; CR2+, subsequent remission.

### Patients in CR1

The 2-year overall survival (OS) was 69% for the MSD group, 65% for the Haplo group, and 71% for the MUD group (p = 0.24, **Figure 1A**, **Table 2**). In multivariable analyses (**Table 3**), donor type was not associated with OS (HR = 1.23, p = 0.39 for Haplo and HR = 1.04, p = 0.85 for URD, compared with MSD).

The two-year DFS rates were 62% for MSD, 56%, for Haplo, and 61% for the MUD group (p = 0.24, **Table 2**, **Figure 1B**). Multivariable analyses confirmed these findings (HR = 1.01, p = 0.98 for MUD and HR = 1.37, p = 0.16 for Haplo).

The two-year relapse rate was 24% for MSD, 20% for Haplo, and 15% for the MUD group (p = 0.03, **Figure 1C**). In the multivariable analysis, compared with MSD, relapse risk was not different for Haplo (HR = 1.05, p = 0.89) nor MUD (HR = 0.68, p = 0.21).

Non-relapse mortality was higher for alternative transplants: 25% for Haplo and 24% for MUD at two years, compared with 13% for MSD (p = 0.28), as shown in **Figure 1D**. Multivariable analysis confirmed these results in absolute terms, but with only a trend for statistical significance for Haplo (HR = 1.72, p = 0.11) compared with MSD.

In univariable analysis, the 2-year cumulative incidence of chronic GVHD significantly differed between the three groups: 44% for MSD, 23% for Haplo, and 31% for MUD (p = 0.013). However, in multivariable analysis, only Haplo had a significantly lower risk of chronic GVHD (HR = 0.55, p = 0.048 for Haplo and HR = 0.96, p = 0.87 for MUD, compared with MSD).

### Patients in CR2+

As shown in **Tables 2** and **3** and **Figure 2**, MUD had better OS and DFS, mainly because of lower relapse rates. NRM and chronic GVHD were not different between the three groups.

## Discussion

Our study, which reflects the outcomes of the early implementation of Haplo with PTCy in a developing country, found that donor type was not associated with OS, DFS, or relapse in CR1. However, NRM was higher with alternative transplants, and chronic GVHD was lower with PTCy-based Haplo. On the other hand, MUD results were significantly superior in patients in CR2+, which may indicate a selection bias among patients in CR2+ who ultimately received HCT.

In patients in CR1, OS, DFS, and relapse, there was no difference between the three groups of donor type, despite a higher NRM with alternative donors. The improved OS and DFS observed in the MUD group in patients in CR2+ were due to a lower relapse rate associated with MUD. Since there is no biological reason to MUD be more effective in CR2+ than CR1, these findings may be influenced by selection bias: identifying, confirming, and scheduling a MUD can postpone a hematopoietic cell transplant (HCT) for several weeks or even months, which can be detrimental for patients with aggressive diseases (survival bias). Atsuta et al. (3) have shown that patients who do not attain HCT must be considered when analyzing transplants from various types of donors. Balduzzi et al. (4) have demonstrated beautifully in practice how longer waiting times paradoxically seem to improve outcomes. Patients with higher-risk diseases may become ineligible for a transplant or die while waiting, thus selecting a population considered to have better outcomes (survival bias). Since bias and residual confounding are more likely explanations for the lower relapse rates observed with MUD compared with MSD and Haplo in patients in CR2+, we can nearly rule out other reasons for these results in our study. Lower relapse rates were limited to CR2+ URD patients, suggesting that a longer waiting time to receive a MUD transplant compared with MSD and Haplo in our country is critical in patients in CR2+. The limited number of centers currently available in the country for hematopoietic cell donation is a major cause of the long delays in obtaining a graft product from the Brazilian Registry of Volunteer Donors of Bone Marrow (REDOME). To rectify this problem, the Brazilian Society of Bone Marrow Transplantation and REDOME met with the Brazilian National Transplantation System in September 2024 to discuss and lay out strategies for improving time delays in graft collection from volunteer HCT donors in the country, but we are not optimistic in the short term.

A lower frequency of MAC regimens in Haplo in our study may have contributed to the higher relapse rate associated with Haplo in CR2+ than MUD. Still, MSD had a similar frequency of MAC regimens, and relapse rates were also higher in CR2+ compared with MUD. Between 2014 and 2018, RIC regimens were widely used in Haplo HCT, and the reported outcomes were quite similar to those of other donor sources. The European Blood and Marrow Transplantation (EBMT) group and others have demonstrated that increasing conditioning intensity may reduce relapse rates after haploidentical transplant for acute leukemias (5–7). However, data remain scarce in this setting. A recent EBMT-matched pair analysis for pediatric AML, which included patients in CR2, reported similar major HCT outcomes for Haplo and MUD (8).

Non-relapse mortality was higher with alternative donors, which might be related to the higher immunosuppressive profile manifested by multiple viral reactivations in Haplo HCT recipients, as previously reported by Brazilian (9), European (10), and North American (11, 12) investigators, which could have been maximized by the usually lower socioeconomic status of patients from developing countries. Anyway, the two-year NRM in patients in CR1 in our study was comparable to those reported in developed countries (10–12). This indicates that MUD and Haplo HCT can be safely performed in these countries.

Chronic GVHD was lower with PTCy-based Haplo, indicating that this is a very effective strategy for GVHD prophylaxis. Most patients in the MUD received ATG (a practice that continues to be supported by a recent Cochrane meta-analysis (13)), while only a few in the MSD received it. Incorporating ATG for PBSC MSD, which has been shown to reduce chronic GVHD in a randomized trial (14), might change this scenario for MSD.

The proportion of Haplo HCT in the country increased from 4% of allogeneic HCT in 2014 to 26% in 2018, and has continued to increase (15). Haplo has already outnumbered MUD, which may reflect fulfilling a previously unmet need, a shift from MUD to Haplo, or a combination of both. We must emphasize intrinsic Haplo *vs* MUD comparisons bias, limited to patients in CR2+, probably played a role, and the expected waiting time of an MUD can hamper access to the transplant itself, when Haplo should be strongly considered.

We also have noticed that more than 50% of transplants were MSD. Currently, developed countries have been reporting 70% of alternative transplants (16). The Brazilian fecundity rate was 2.1 in 2002 (data source: https://datatopics.worldbank.org/world-development-indicators/), meaning that only 26% of the patients born during that fecundity rate would have an MSD ( $Probability\left(MSD\right)=1-{\left(1-24\%\right)}^{\left(Fecundity\;Rate-1\right)}$ ). Indeed, this number is compatible with our children’s data. For comparison, the fecundity rate in 1980 was 4.04 (meaning a Probability(MSD) of 56%), and adult patients may have been born in larger families or are being deferred from alternative transplants because of age (the upper age limit for coverage of URD in Brazil was 65 years during the study period). Our study was not designed to address family sizes as a potential contributor to high rates of MSD transplants.

This study has several limitations. It is a retrospective study. Nonetheless, using the registry to assess real-world data in Brazil appears to be the most feasible approach, facilitating a study that might not have been conducted otherwise. However, data access is limited regarding measurable residual disease before transplant, the presence of anti-donor specific antibodies for Haplo, the time from diagnosis to transplant, the occurrence and severity of acute GVHD, and the use of prophylactic donor lymphocyte infusions. However, acute GVHD has three outcomes: patient dies, recovers, or evolve to chronic GVHD. All those three outcomes were captured in our study. Furthermore, the conditioning selection algorithms in Haplo and the GVHD prophylaxis regimens used between 2014 and 2018 may not reflect current practices. Also, the majority of the patients were MSD in CR1, and unexperienced readers may overestimate the power of our study. Still, this study represents a large cohort of HCT in Brazil, supported by the CIBMTR. It may serve as a trampoline to enhance national studies in countries without an established registry.

In summary, our study suggests MUD and Haplo HCT donors are acceptable options for patients lacking MSD in developing countries like ours. However, comparisons of MUD and Haplo have intrinsic bias, especially in CR2+, and the results of observational studies like ours should be interpreted with caution in this population. Enhancing MUD HCT timing in CR2+ should be a goal for REDOME. We also demonstrated the feasibility of the collaboration between SBTMO and CIBMTR in conducting local Brazilian studies. Our study may also encourage countries without an established registry to collaborate similarly. A paired study using more recent prospective data from the SBTMO is ongoing.

## Data Availability

The raw data supporting the conclusions of this article will be made available by the authors, without undue reservation.
