# Supplementary material for: Haploidentical, matched-related, and matched-unrelated hematopoietic cell transplant for acute leukemias in the early years of haploidentical transplant implementation in a developing country with a large unrelated donor registry
Source: Front Oncol. 2025 Apr 15;15:1584631. doi: 10.3389/fonc.2025.1584631 (PMC12037578; doi:10.3389/fonc.2025.1584631)

# **Supplemental material**

**Supplemental Table 1. Participating centers, in alphabetic order**

| Participating centers | City |
| --- | --- |
| A.C. Camargo Cancer Center | Sao Paulo |
| Associacao Hospitalar Moinhos de Vento | Porto Alegre |
| Beneficiencia Portuguesa de Sao Paulo | Sao Paulo |
| Bio Sana's Servicos Medicos | Sao Paulo |
| Centro de Hematologia de Hemoterapia de Santa Catarina | Florianopolis |
| Grupo de Apoio ao Adolescente e a Crianca com Cancer | Sao Paulo |
| Hospital Amaral Carvalho | Jau |
| Hospital de Cancer de Barretos | Barretos |
| Hospital Israelita Albert Einstein | Sao Paulo |
| Hospital Samaritano | Sao Paulo |
| Hospital Sirio-Libanes | Sao Paulo |
| Instituto de Cardiologia do Distrito Federal | Brasilia |
| Instituto Nacional de Cancer | Rio de Janeiro |
| Universidade Estadual de Campinas | Campinas |
| Universidade Federal de Minas Gerais | Belo Horizonte |
| Universidade Federal do Ceara | Fortaleza |
| Universidade Federal do Parana | Curitiba |
| Universidade Federal do Rio Grande do Norte | Natal |
| Universidade Federal do Rio Grande do Sul | Porto Alegre |

Table 2. Variables tested in all multivariable analyses

| Variables tested |
| --- |
| Donor type |
| Age |
| Children (<18 y/o) or adult |
| Sex |
| HCT-CI |
| KPS |
| Disease |
| Donor sex |
| Sex match |
| Graft (BM or PBSC) |
| Conditioning (NMA, RIC, or MAC) |

Table 3. Variables included in the multivariable analyses

| Outcome | Controlled for: |
| --- | --- |
| **CR1** |  |
| Overall survival | KPS, conditioning |
| Disease-free survival | <18 y/o, KPS, graft |
| Relapse | KPS |
| Non-relapse mortality | KPS, conditioning |
| Chronic GVHD | <18 y/o, disease, sex match |
| **CR2** |  |
| Overall survival | Age, disease |
| Disease-free survival | Sex match |
| Relapse | <18yo, sex match, disease |
| Non-relapse mortality | <18yo, disease, conditioning |
| Chronic GVHD | Sex match |

Table 4. Causes of death

|  | Haplo, CR1 | Haplo, CR2+ | MSD, CR1 | MSD, CR2+ | MUD, CR1 | MUD, CR2+ |
| --- | --- | --- | --- | --- | --- | --- |
| Total | 60 | 56 | 248 | 90 | 79 | 62 |
| Alive | 36 (60%) | 24 (42.9%) | 159 (64.4%) | 27 (31%) | 53 (67.1%) | 38 (61.3%) |
| Primary disease | 8 (13.3%) | 20 (35.7%) | 46 (18.6%) | 36 (41.4%) | 6 (7.6%) | 13 (21%) |
| Graft failure | 0 | 1 (1.8%) | 2 (0.8%) | 0 | 0 | 1 (1.6%) |
| GVHD | 2 (3.3%) | 3 (5.4%) | 7 (2.8%) | 3 (3.4%) | 5 (6.3%) | 2 (3.2%) |
| Infection | 7 (11.7%) | 6 (10.7%) | 20 (8.1%) | 12 (13.8%) | 10 (12.7%) | 5 (8.1%) |
| IPn | 3 (5%) | 0 | 2 (0.8%) | 0 | 1 (1.3%) | 0 |
| ARDS | 1 (1.7%) | 1 (1.8%) | 2 (0.8%) | 0 | 1 (1.3%) | 0 |
| Organ failure | 3 (5%) | 1 (1.8%) | 4 (1.6%) | 2 (2.3%) | 0 | 2 (3.2%) |
| Organ toxicity | 0 | 0 | 1 (0.4%) | 0 | 0 | 0 |
| Secondary malignancy | 0 | 0 | 0 | 2 (2.3%) | 0 | 0 |
| Hemorrhage | 0 | 0 | 2 (0.8%) | 2 (2.3%) | 0 | 1 (1.6%) |
| Vascular | 0 | 0 | 1 (0.4%) | 1 (1.1%) | 2 (2.5%) | 0 |
| Other | 0 | 0 | 1 (0.4%) | 2 (2.3%) | 1 (1.3%) | 0 |

Figure 1. Cumulative incidence of neutrophil and platelet engraftment


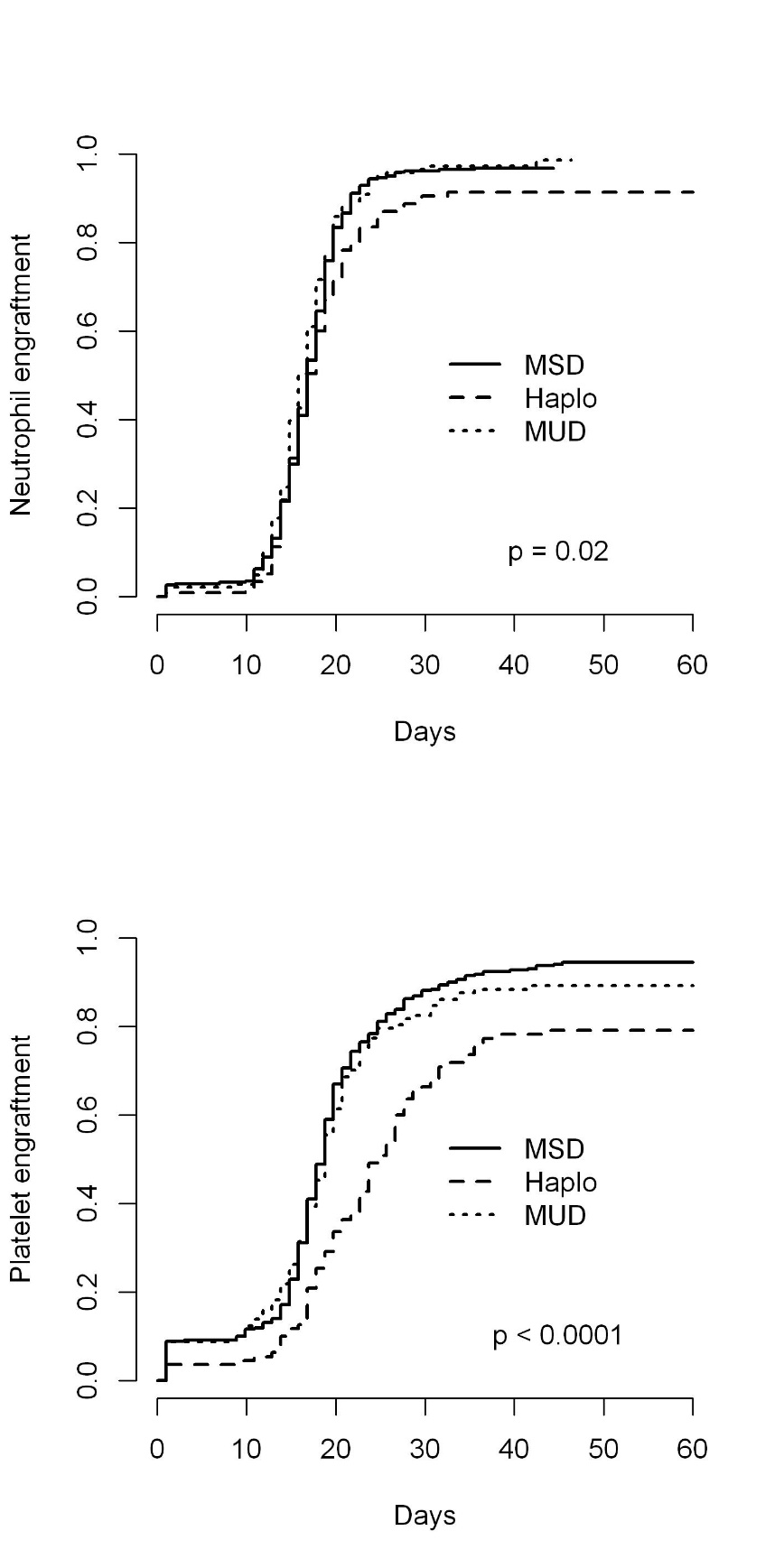

Supplement: Supplementary file 1 [file DataSheet1.docx]
